# Sustainable use zoning of land resources considering ecological and geological problems in Pearl River Delta Economic Zone, China

**DOI:** 10.1038/s41598-019-52355-7

**Published:** 2019-11-05

**Authors:** Lin Gao, Chuanming Ma, Qixin Wang, Aiguo Zhou

**Affiliations:** 1 School of Geological Survey, China University of Geosciences, Wuhan, China; 2 School of Environmental Studies, China University of Geosciences, Wuhan, China; 3 China Railway Design Corporation, Tianjin, 300251 China; 4 School of Environmental Studies, China University of Geosciences, Wuhan, 430074 China

**Keywords:** Urban ecology, Sustainability

## Abstract

The Pearl River Delta Economic Zone is one of the fastest growing areas of China’s social and economic development. However, the contradiction between people and land, the deterioration of ecological environment and the damage of urban ecological security have become more serious problems. In previous studies there was single land utilization type in small-area and the evaluation method was not suitable to large areas, this study proposes a new method. Firstly, the study implements ecological land zoning from assessing the importance of ecosystem services functional in four aspects: biodiversity, water conservation, soil conservation and coastal protected zone. Then, the suitability evaluation index system of agricultural and construction land is established from the geological environment perspective, and introduces variable weight-analytical hierarchy process-comprehensive index model to evaluate the suitability of agricultural land and construction land. Re-zoning the type of land that has a special effect on the socio-economic, the mining land, protected area of geological relics and groundwater resources, respectively. Finally, considering the actual condition use status and suitability distribution of land, the results of comprehensive zoning of land utilization is got. The results of this study can provide some geological basis for the future land utilization zoning.

## Introduction

With the rapid development of China’s economy and urbanization process, the land utilization types have changed dramatically of Pearl River Delta Economic Zone^1^. Due to the land utilization structure and layout are not rational, construction land occupies large-scale agricultural land, leading to a sharp decline in agricultural land and ecological degradation of land resource^2,3^, which has heavily exploited natural resources and damaged geological environment conditions. This has led to a series of geological environmental problems, such as groundwater pollution, soil heavy metal pollution, soil erosion, ecosystem degradation, and loss of biodiversity^4,5^. Targeting these problems, some researchers have proposed to re-zoning the land utilization types of cities in the Pearl River Delta Economic Zone^6,7^. But previous studies concentrated in a certain city or a small area, lacked the research on land utilization in the area of space^3,8,9^. With the continuous innovation of land utilization zoning research methods, land utilization zoning research presents a new trend of modernization, stratification and integration^10–15^. Researchers began using GIS platform, zoning by different methods^11,12,16^ based on measurement data and weighting criteria, apply multi-factor models with the help of IDRISI GIS software, researched the applicability of commercial agriculture, small-scale agriculture, urban development, nature conservation, respectively. Ceballos-Silva, A.^17^ used the pairwise comparison method to determine the weight, and used the 0–5 scale to evaluate the results, then conducted the land suitability evaluation of particular crop; Giap, D.H.^18^ used Analytic Hierarchy Process (AHP), under taking into account constraints, used the relative importance of specific factors to measure and assess applicability; Liu, Y.^19^ developed an adaptive fuzzy inference method for evaluation of agricultural land in Hubei Province. However, the area of above study is small, and most of the research zoning methods are directed to a single land type, which does not meet the requirements of overall zoning. Oliver T. Hogg^20^ proposing to analyze state of marine protected area through landscape mapping data in southern Antarctica. Soumendra N. Bhanja^21^ achieved the effectiveness of India’s water management policy by tracking data on groundwater in parts of India. In the few zonings of virous land types, the functional applicability of virous land types was neglected, and a single evaluation method was used to virous land types zoning^22–24^. And more attention was paid to the developmental forces of human social and economy, missing from the perspective of basic geological environment conditions consider the land utilization types, resulting in conflicting land resource, further deterioration of the ecological environment^25,26^. Therefore, this paper takes the Pearl River Delta Economic Zone as an example, based on the premise of ensuring regional ecological and environmental security, evaluation the ecological land by objective weighting method. Starting from the geological environment elements associated with land utilization types, in order to solve the problem that the weight of the evaluation factors constant in the evaluation process, the partial decision results are greatly deviated, the evaluation of agricultural land and construction land by variable weight- analysis hierarchy process-comprehensive index method(VWAHP-CIM)^11,13,16,18,27,28^.

## Study Area

**Figure 1:**
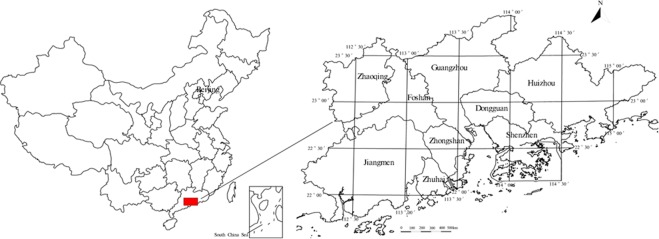
The Location and Scope of the study area.

**Figure 2:**
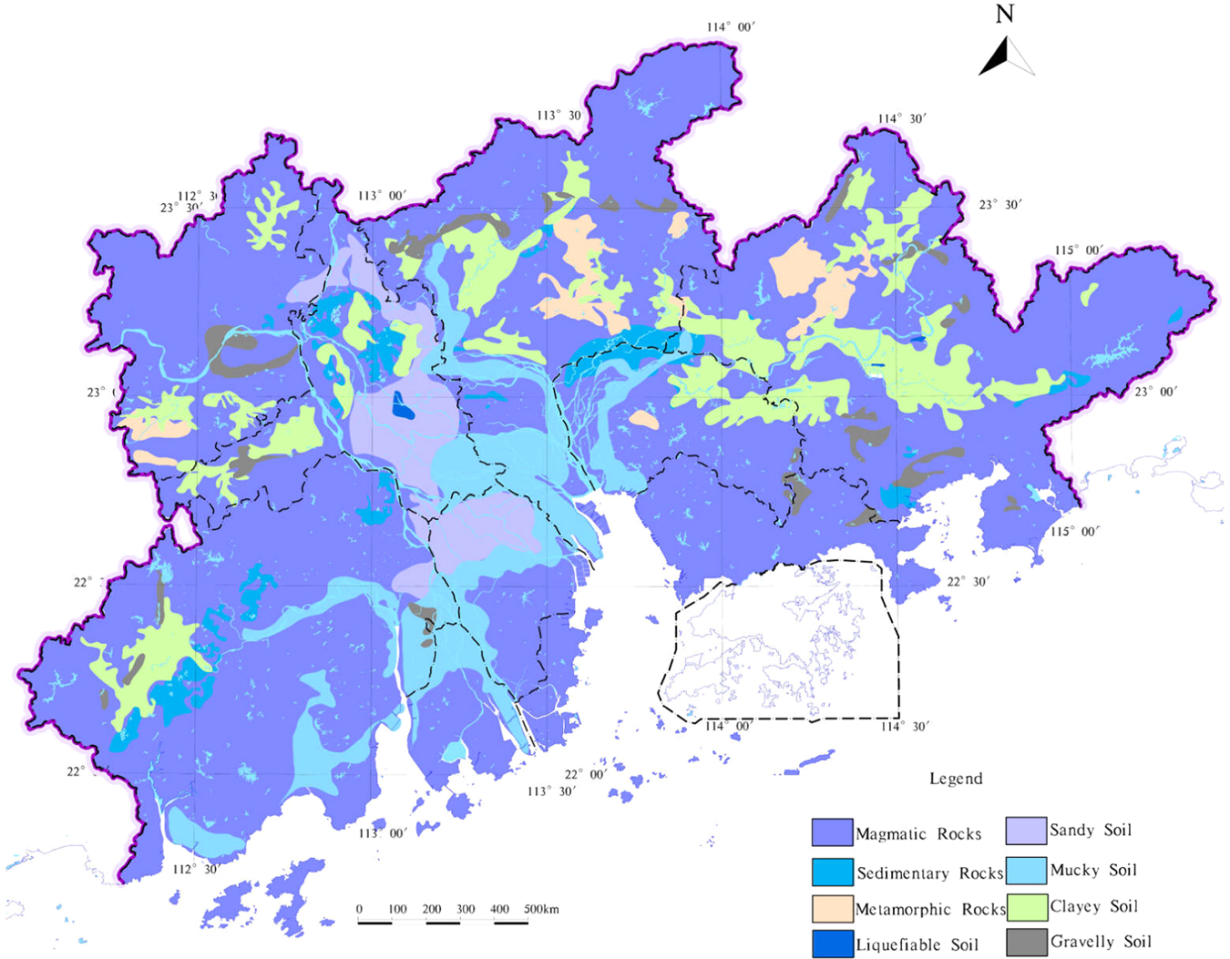
The engineering geology of the study area.

The study area - the Pearl River Delta Economic Zone is located east longitude 111°59′42″–115°25′18″ and latitude 21°17′36″–23°55′54″, with a total area of about 55407 km^2^, including 9 administrative regions (Fig. 1). In the study area, the terrain is flat and the topography is small. From the oldest Mesoproterozoic metamorphic rocks to the latest Quaternary loose debris deposition have exposed. Most of the study area are covered by Quaternary and magmatic rocks are widely exposed to the surface, which account for about 30% of the study area. In the river network area and the coastal plain area, the surface is covered with silt clay, which has the characteristics of low shear strength, high compressibility and low bearing capacity (Fig. 2). Local presence seawater intrusion in the study area, which forms different degrees of salt water^7,29^. There are geological hazards and environmental problems such as collapse, debris flow, land subsidence, groundwater pollution and soil heavy metal pollution^30,31^.

## Methods

**Figure 3:**
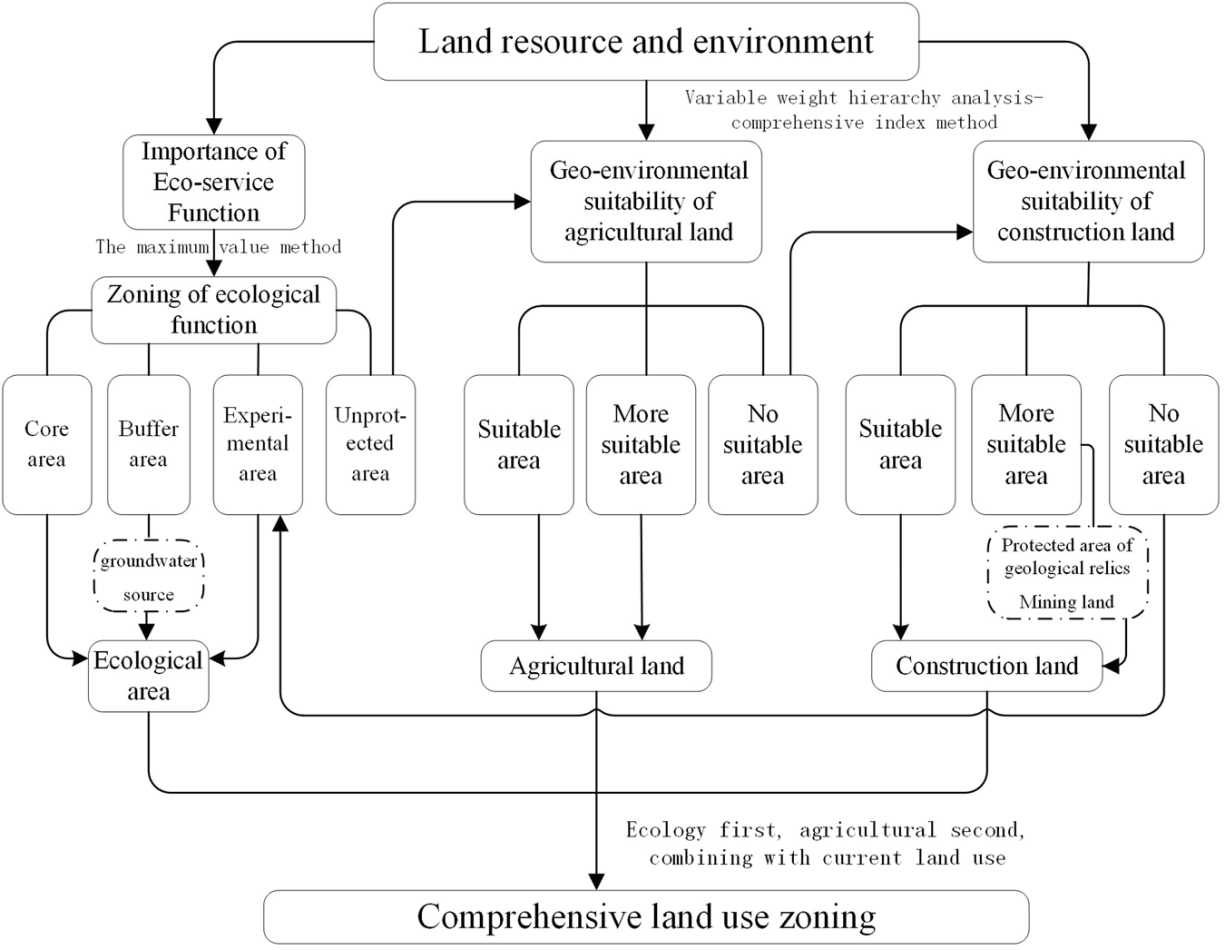
Flowchart of land utilization zoning in the study area.

Flowchart of this study is shown in Fig. 3. As illustrated in Fig. 3, the maximum value method was used to determine the importance of ecological service functions and the variable weight hierarchy analysis-comprehensive index method was used to the agricultural land and the construction land suitability, respectively. Special types of land utilization within the dotted frame has been reserved.

In order to ensuring the ecological security in the area, the study analyzes ecological security risk of the study area, and establishes an evaluation index system for the importance of ecological service functions comprising different single factors. With the support of MapGIS platform, the importance of each single ecological factor is separately segmented, and each single factor ecological service importance zoning map is superimposed. According to the objective weighting^32,33^ Eq. (1), the importance degree of the superimposed evaluation unit is determined, and the ecological protection zoning result of the study area is obtained.

1 $$EI=MAX\{E{I}_{1},E{I}_{2},E{I}_{3},\cdots \,\cdots E{I}_{i}\}$$

where *EI* is the ecological importance of the evaluation unit; *EI*_*i*_ is the importance of each single factor ecological service.

From the perspective of geological environment, the suitability evaluation index systems which associated with agricultural land and construction land are established, respectively, and use AHP to determine the basic weight of evaluation index. Then use Eq. (2), the evaluation index forms orderly arrangement state according to the grouping of hierarchical structure, with 1–9 relative scale quantitative calculation^11^, through the comparison of the two indicators of the relative importance, the basis weight of each index is calculated. Then introduce the variable weight theory (VWT)^28^ to correct the basic weight Eqs (3) and (4) and solve the deviation of partial decision result in the weighted sum, so that the evaluation result is closer to the study reality.

2 $${M^{\prime} }_{ij}=\frac{{M}_{ij}-Mi{n}_{j}}{Ma{x}_{j}-Mi{n}_{j}}$$

3 $${K}_{ij}=\{\begin{array}{cc}1 & {S}_{ij} > {A}_{j}\\ {e}^{-\theta ({S}_{ij}-{A}_{j})} & 0 < {S}_{ij} < {A}_{j}\end{array}$$

4 $${S^{\prime} }_{ij}=\frac{{S}_{ij}{K}_{ij}}{\mathop{\sum }\limits_{i=1}^{n}{S}_{ij}{K}_{ij}}$$

where ${M^{\prime} }_{ij}$ is the score of the j index of the i evaluation unit after normalization, *M*_*ij*_ is the actual score of the j indicator of the i evaluation unit, *Max*_*j*_ and *Min*_*j*_ represent the maximum and minimum value of the j index actual score, *K*_*ij*_ represents the weight correction coefficient of the j indicator of the i evaluation unit, and *θ* represents the degree of penalty of the variable weight. The greater the value is, the better the penalty effect is, *A*_*j*_ indicates the critical value of the normalized value of the j indicator score after the descending order of two thirds, ${S^{\prime} }_{ij}$ represents the weight correction value of the j index of the i evaluation unit, and *S*_*ij*_ represents the basis weight of the j index of the i evaluation unit.

With the support of MapGIS platform, based on the suitability index grading standard of agricultural and construction land, the suitability evaluation of single index is carried out, and the new evaluation unit obtained from the superimpose the single index result, zoned by comprehensive index method (CIM) Eq. (5) for the agricultural land and the construction land suitability, respectively. Then, according to the suitability grading standard of agricultural and construction land, the results of the distribution of agricultural and construction land are obtained, respectively.

5 $${P}_{i}=\mathop{\sum }\limits_{i=1}^{n}{S^{\prime} }_{ij}{M}_{ij}$$

where *P*_*i*_ represents the suitability score of i evaluation unit.

From the zoning results of agricultural and construction land, extract the land that play a special impact on the social and economic development, protected area of geological relics, mining land, protected area of groundwater resources, respectively, the zoning results of land utilization are obtained according to the determination standards.

Then comparing the distribution of land utilization status in the study area, considering the actual condition use status and suitability distribution of land, finally the comprehensive zoning of land utilization in the study area is put forward.

### Zoning processes

#### Determination of land utilization types

The ecological land has the function of protecting the city’s ecological system, biological habitat and improving the quality of life of the residents. The quantity and spatial distribution of ecological land will have an important influence on the urban ecological security^34,35^. Agriculture is the basic material guarantee of human survival and development, the rationality of its use is related to the sustainable development of human society. Construction land not only provides a basic space for human living environment but also carries a variety of human economic activities, which has a direct relationship on the size of urban land area and economic benefits of output. Based on the above considerations, determine the land resource function of the Pearl River Delta Economic Zone as: ecological land, agricultural land and construction land.

#### Ecological land zoning

Ecosystem provides human beings with a variety of services in both natural and subsistence environments. The sustainable supply of these services is the basis for economic and social development^36–38^. Due to the different geological environmental conditions, natural resources and living environments vary from region to region, it has been confirmed by previous studies^39^. For the Pearl River Delta Economic Zone, the service function of the ecosystem maintains the natural resource functions of biodiversity and water conservation, and provides the living environment functions of soil conservation and coastal protection.

#### Evaluation index system of ecosystem services functional importance

**Figure 4:**
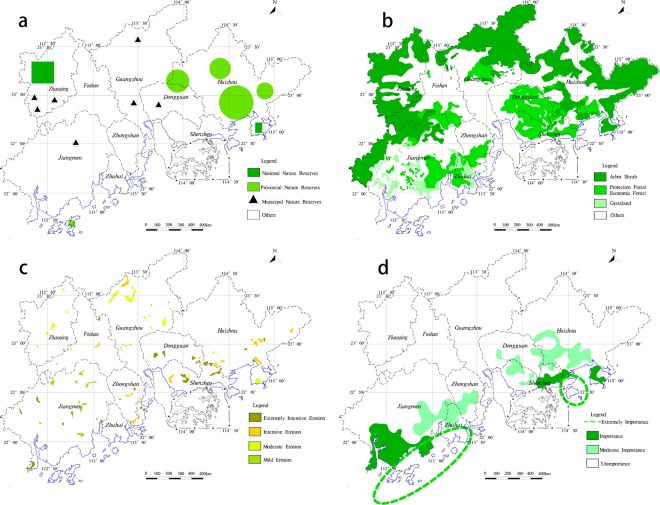
Each index important degree of ecosystem services functional. (**a**) Biodiversity importance; (**b**) Water conservation; (**c**) Soil erosion; (**d**) Coastal protected.

Biodiversity is the core of the ecosystem^40^, which supports all types of ecosystem service. The biodiversity will not only directly affect the ecosystem services function, but also one of the most critical indexes that constrain exerting of ecosystem services function^41–44^. The research selects natural reserves at different administrative levels as the basis for dividing the important of biodiversity conservation (Fig. 4a). Water conservation function is an important component of ecological function, its size affects the development of ecosystem^45^. Decline in water conservation can lead to the depletion of water resources^46^, which may have a devastating impact on ecosystem stability. The different vegetation types have different abilities for water conservation. According to different vegetation types, it combines the actual vegetation types in the study area to distinguish important water conservation function (Fig. 4b).

Soil erosion not only causes the loss of water and soil resources and the destruction of land productivity, but also the harm to human survival^47–49^. Due to soil erosion, ecological problems such as soil fertility decline have exacerbated the fragility of ecosystems and will affect human production and life (Fig. 4c). Coastal ecosystems have a protective effect against typhoons, waves and erosion^50^. Scientific and effective delineation of coastal zones is not only a green barrier against various threats but also plays an important role in maintaining ecological balance (Fig. 4d). According to the indicators grading standards of ecosystem services functional (Table 1), the important ecosystem services functional of each evaluation index is classified different levels.

**Table 1:** The evaluation index of importance of ecological service function.

Evaluation index	The important degree of ecological service function	Classification standards	Score
Biodiversity conservation (*EI*_bq_)	Extremely importance	National nature reserve	100
	importance	Provincial nature reserves	75
	Moderate importance	municipal nature reserves	50
	unimportance	Others	25
Water conservation (*EI*_sy_)	Extremely importance	Arbor Shrubs	100
	importance	Protected forest and Economic forest	75
	Moderate importance	Grassland	50
	unimportance	Others	25
Soil erosion ($E{I}_{{\rm{sl}}}$)	Extremely importance	Extremely intensive erosion	100
	importance	Intensive erosion	75
	Moderate importance	Moderate erosion	50
	unimportance	Mild erosion	25
Coastal zone protected function (*EI*_hd_)	Extremely importance	Central Dayawan area and Daguanghai bay area	100
	importance	Coastal zone and the island	75
	Moderate importance	Coastal forest	50
	unimportance	Others	25

#### Evaluation of importance of ecosystem services functional

**Figure 5:**
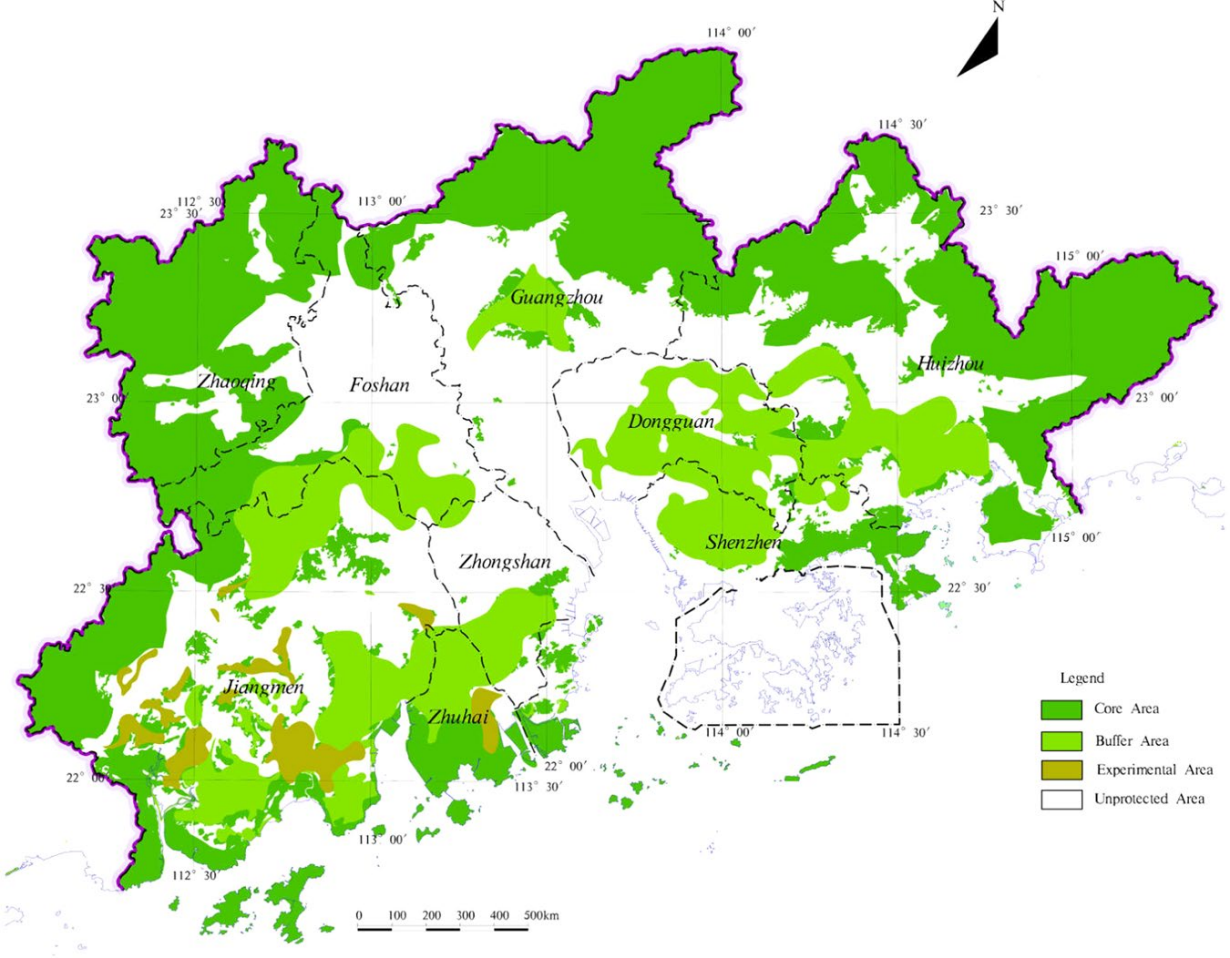
The ecological protection zoning in the study area.

Based on the MapGIS platform, the ecological service function importance zoning of each evaluation index is superimposed. Using Eq. (1) and according to Table 2, level of unit evaluation is determined, so that the ecological protected zones are obtained (Fig. 5).

$$EI=Max\{E{I}_{bd},E{I}_{sy},E{I}_{sl},E{I}_{hd}\}$$

where EI is the ecological importance of unit, *EI*_bd_ is biodiversity conservation, *EI*_sy_ is water conservation, *EI*_sl_ is soil erosion, and *EI*_hd_ is coastal protected function.

**Table 2:** The classification standards of ecological protection zoning.

Classification standards	EI
Core area	100
Buffer area	75
Experimental area	50
Unprotected area	25

### Agricultural land zoning

The quality of agricultural geology and environmental conditions affect the safety of crops. Soil as a product of geological processes, which provide the growth of crops with the basic carrier conditions. Groundwater is an essential element of the growth of crops, and geomorphological conditions affect the suitability of crops.

#### Selecting evaluation index system of agricultural land suitability

**Figure 6:**
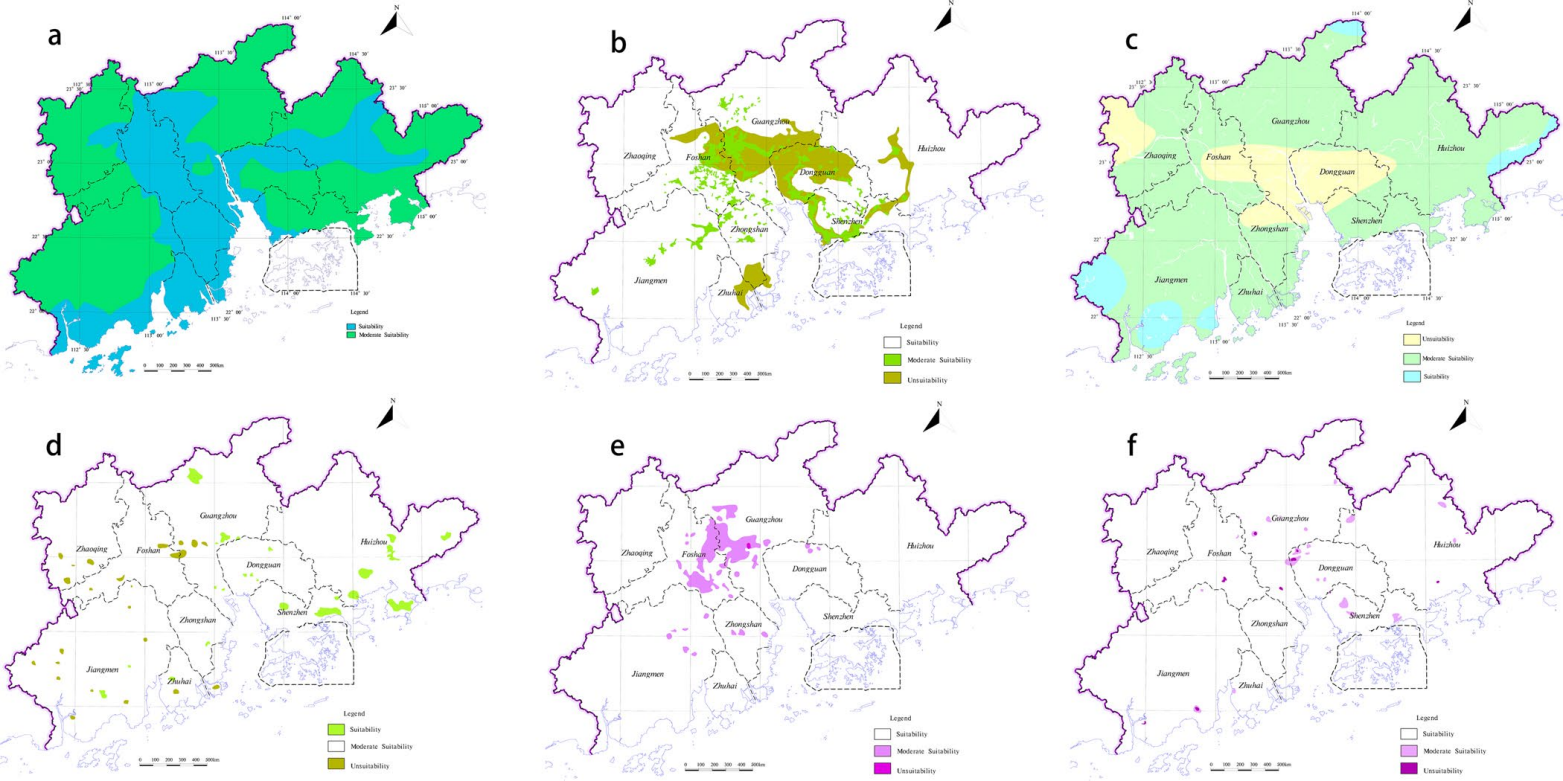
The agricultural land suitability evaluation of each index. (**a**) Landscapes; (**b**) Rainfall; (**c**) Groundwater pollution; (**d**) Arsenic; (**e**) Mercury; (**f**) Chromium.

In the evaluation index system of agricultural land, the landform is defined as the form and appearance of the surface ups and downs, because each landform has the characteristics of terrain slope, sediment soil and hydrological nature^51^. Therefore, the landform conditions determine the basic direction and layout of agricultural land utilization (Fig. 6a). The quality of groundwater will not only affect the quality of agricultural products but also play an important role in the consumption rate of soil organic matter and the capacity of soil regeneration^52^ (Fig. 6b). Precipitation is the main limiting index for the growth of crops (Fig. 6c). If the crop roots absorption heavy metal elements in the soil, thus it will affect human health. Combined with the actual soil conditions of the study area, and select the abnormal heavy metal elements of As (Fig. 6d), Hg (Fig. 6e) and Cd (Fig. 6f) as the evaluation index^30,53^. For most study areas, the increase in precipitation is appropriate, which is conducive to the growth and development of crops and improve the regional agricultural productivity. Therefore, in the agricultural land suitability evaluation, select the geological indexes that affect the growth of crops as the evaluation index (Table 3).

**Table 3:** Evaluation index of suitability of agricultural land.

Criterion	Constant weights	Variable weights	Evaluation index	Suitability (5)	Moderate Suitability (3)	Unsuitability (1)	Constant weights	Variable weights
Natural geography	0.252	0.259	Geomorphology	Plain	Hills	Mountain	0.121	0.125
			Annual precipitation (mm/a)	>2200	1600–2200	<1600	0.131	0.134
Water quality	0.233	0.224	Groundwater pollution	Uncontamination	Slight contamination	Heavy contamination	0.233	0.224
Soil quality	0.515	0.517	Arsenic (PPM)	<40	40–80	>80	0.175	0.174
			Mercury (PPM)	<0.5	0.5–3	>3	0.171	0.172
			Chromium (PPM)	<15	90–300	>300	0.169	0.171

### Construction land zoning

The relationship between geological environment and the construction land is the complex interaction. On the one hand, urban safety is the basic condition of human survival, and the suitability of geological environment conditions determine the safety of urban construction. Besides, urban construction process is also transforming the surrounding geological environment, which may lead to the deteriorate of regional geological conditions or exacerbate occur adverse geological effects, so it will affect the survival safety.

#### Evaluation index system of construction land suitability

**Figure 7:**
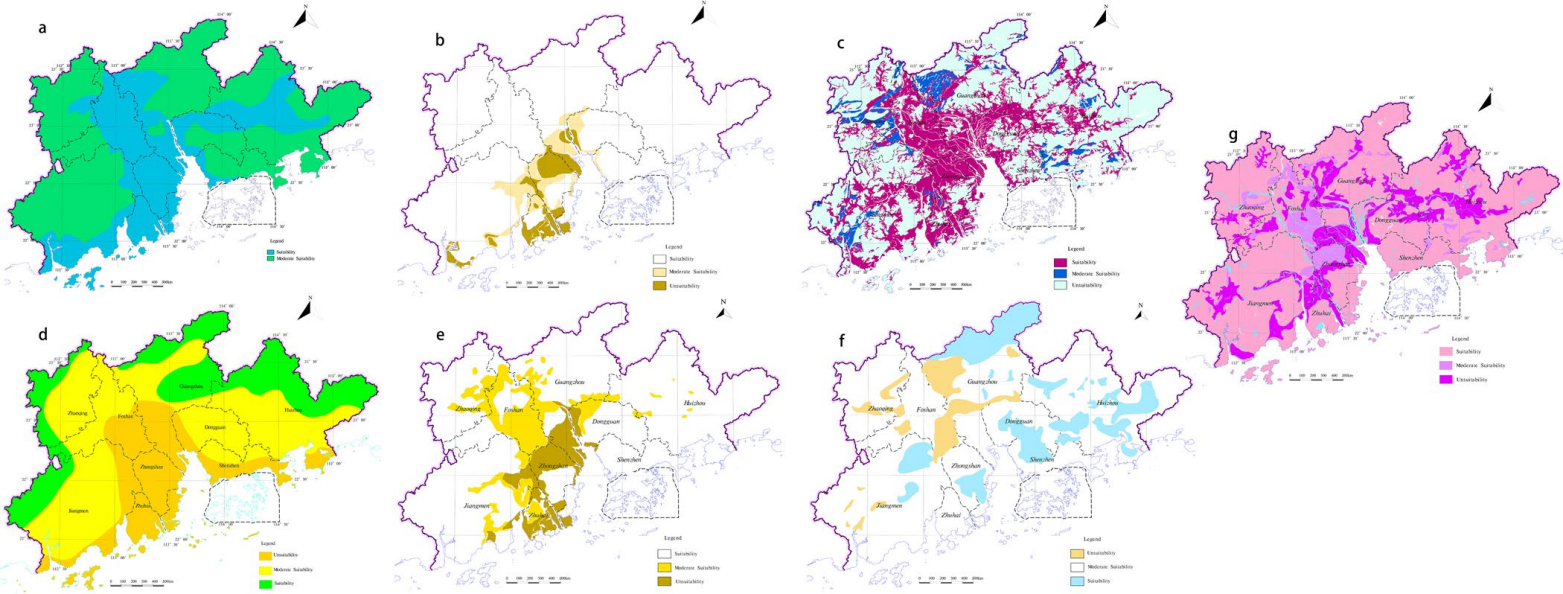
The construction land suitability evaluation of each index. (**a**) Landscapes; (**b**) Groundwater; (**c**) Salt water; (**d**) Fracture; (**e**) Ground subsidence; (**f**) Foundation soil; (**g**) Geohazards probability.

In the evaluation index system of construction land suitability, the landforms affect the determination of urban location, selection of urban construction site, zoning layout, engineering facilities, layout of construction and other aspects. Because the terrain is flat, which is favorable to develop urban construction, and the terrain slope of the larger hills and mountain areas, where difficulty of urban construction has increased significantly, so the landforms has an important impact on construction land zoning (Fig. 7a). The level of groundwater mineralization reflects the corrosive nature of anions and cations for underground infrastructure to affect the safety and stability of the buildings (Fig. 7b). The area where karst water distributed is a high prone area for ground collapse, which endangers the safety of surface buildings (Fig. 7c). The fault condition reflects the structural stability of the area. Owing to fault slip, buildings on active faults after the earthquake, which is more prone to damage (Fig. 7d). Ground subsidence can cause the foundation to be disturbed and destroyed, which can cause the buildings to tilt, wall cracking, and even affect the normal use (Fig. 7e)^54^. The higher the degree of geological hazards, the greater the likelihood of damage to buildings (Fig. 7f). The type of foundation soil is directly related to the stability of the foundation, the clay soil is prone to shear failure under the action of the load, and the liquefiable soil can cause the foundation failure during the earthquake, muddy soil with stand lower loads, which is more prone to ground subsidence (Fig. 7g). Therefore, from the perspective of geological environment which affects the safety of urban land construction, selects the suitability evaluation index system of construction land (Table 4).

**Table 4:** The evaluation index of suitability of construction land.

Criterion	Constant weights	Variable weights	Evaluation index	Suitability (5)	Moderate Suitability (3)	Unsuitability (1)	Constant weights	Variable weights
Natural geography	0.113	0.115	Geomorphology	Plain	Hills	Mountain	0.113	0.115
Hydrogeology	0.263	0.264	Type of groundwater	Pore water	Interstitial water	Karst water	0.117	0.119
			Saline water (TDS)	<1 g	1–10 g	>10 g	0.146	0.145
Engineering Geology	0.488	0.484	Fracture	No development	General development	Development	0.177	0.173
			Ground subsidence	Area of no ground subsidence	Area of soft soil distribution	Area of ground subsidence prone	0.163	0.161
			Foundation soil	Rocks	Gravel and sand	Mucky soil and cohesive soil	0.148	0.150
Environmental geology	0.136	0.137	Geological hazard probability	Low-probable geological hazard	Mid-probable geological hazard	High-probable geological hazard	0.136	0.137

#### Zoning and evaluation of agricultural land and construction land

**Figure 8:**
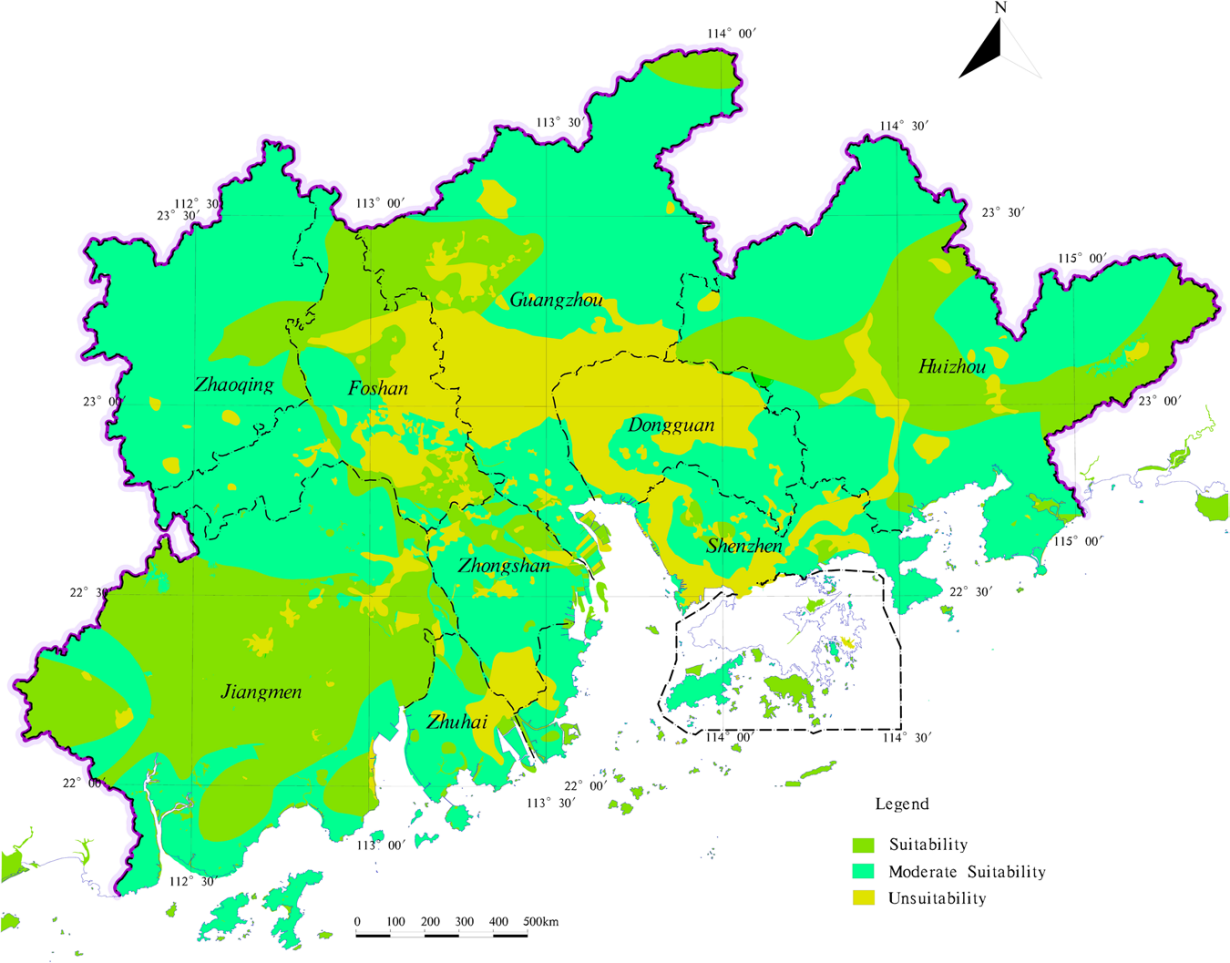
The zoning of agricultural land suitability evaluation in the study area.

**Figure 9:**
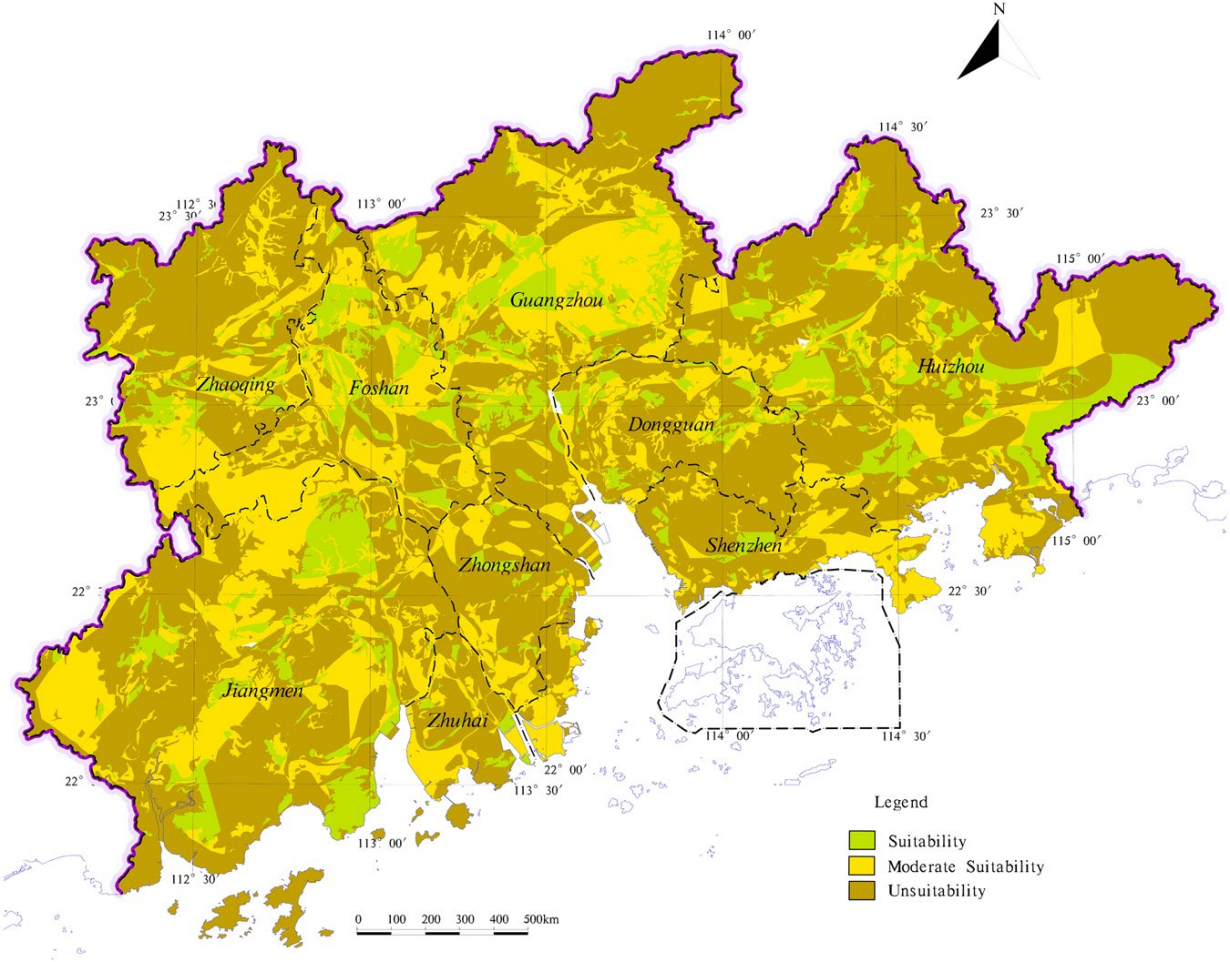
The zoning of construction land suitability in the study area.

Based on the present conditions of evaluation index of suitability, according to the evaluation index grading standard of agricultural land, construction land (Tables 3, 4), with the support of MapGIS platform, the research superimposes each suitability index zoning results (Figs 6, and 7). And use the attribute boundary of each evaluation index after superimposition serves as the evaluation unit. Then introduce the VWAHP-CIM calculate each evaluation unit score of suitability. According to the land suitability evaluation type of grading criteria (Table 5), the results of land suitability zoning are got in the study area (Figs 8, and 9).

**Table 5:** The classification standards of suitability evaluation

Suitability score	<2	2–4	>4
Suitability level	Unsuitability	Moderate Suitability	Suitability

### Special types of land utilization

Based on the actual conditions of land utilization in the study area, the mining land, protected area of geological relics and protected area of groundwater resources are extracted separately, which have special effects on local social economic development and ecological security.

**Figure 10:**
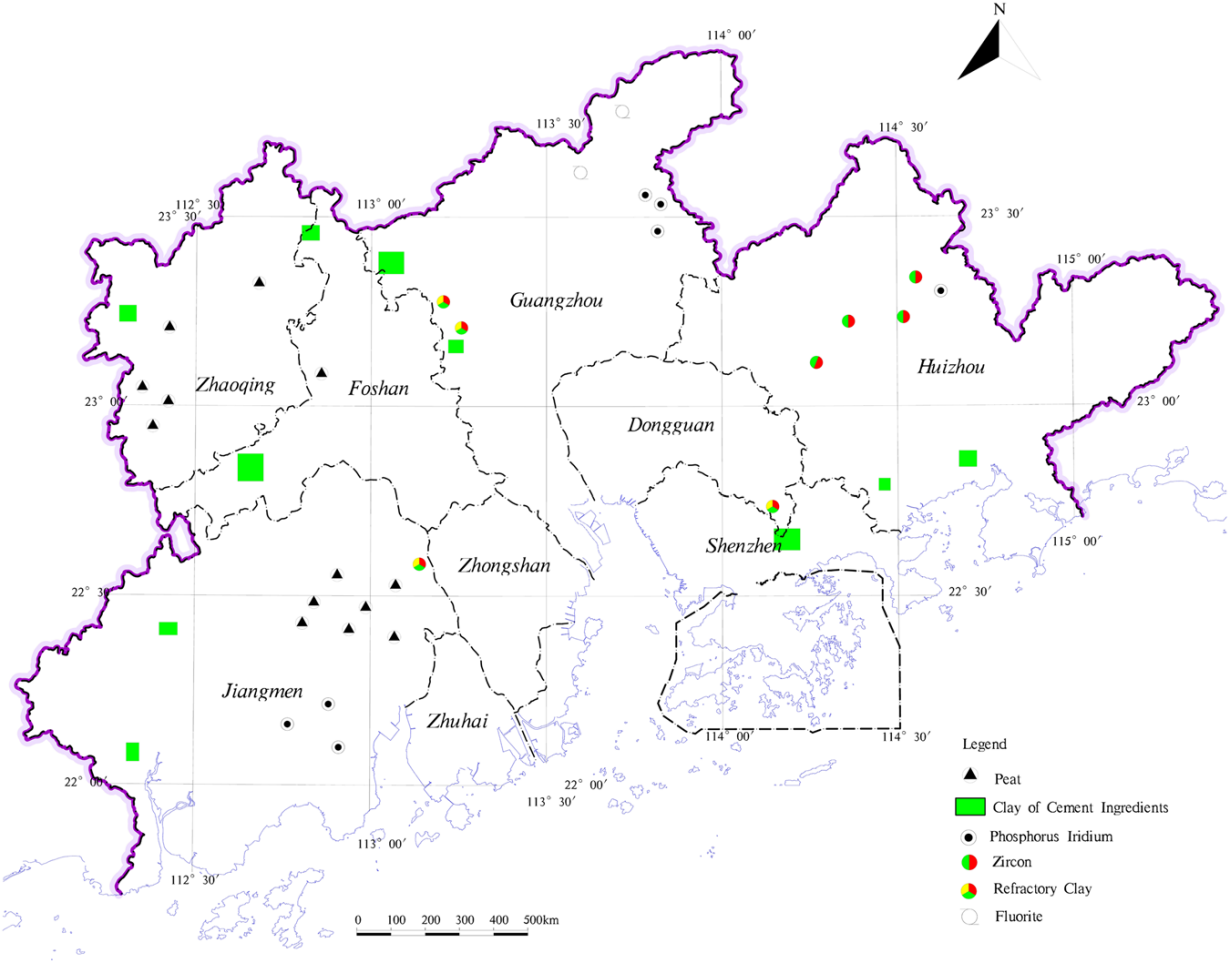
The distribution of mineral resources in the study area.

**Figure 11:**
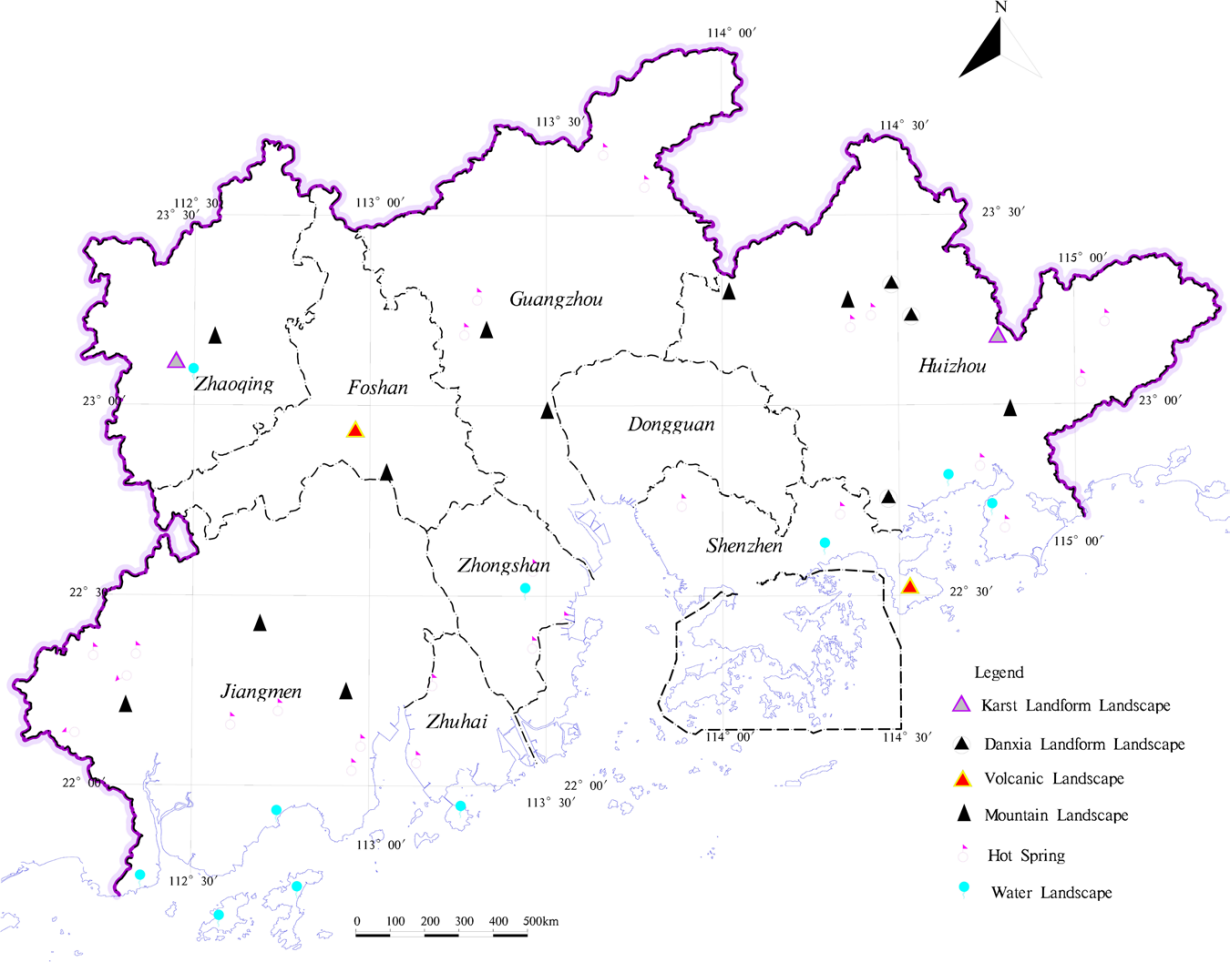
The distribution of geological relics in the study area.

**Figure 12:**
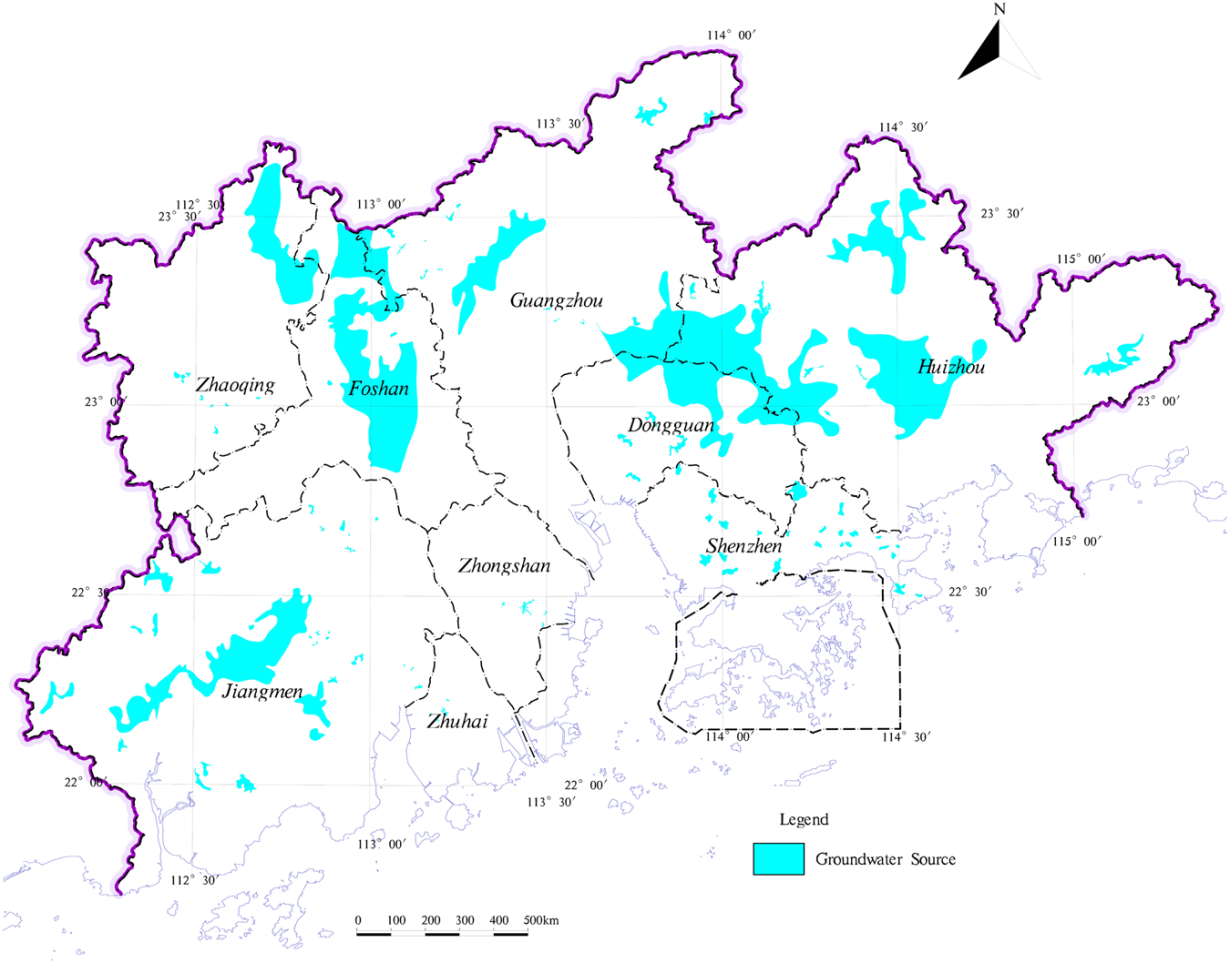
The distribution of groundwater source field in the study area.

Mineral resources and land resources are essential for human survival and social development of the means of production. The exploitation of mineral resources stimulates the local socio-economic development, meanwhile, the exploitation of mineral resources damages the arable land resources. The non-metallic mineral resources are the main mineral resources in the study area. And although the study area has a large variety of minerals, yet the scale of mineral resources is smaller, which is mostly distributed in hilly areas outside the study area where the ecological environment more superior (Fig. 10). Furthermore, mineral exploitation is relatively great difficulty and causes large damage to ecological environment. Geological relics are non-renewable, geological and natural heritage, with important scientific value and social value. Geological relics not only promote tourism development but also play an important role in improving the quality of regional ecological environment^55^. As the destruction of geological relics by exploitation of mineral resources and other human indexes are becoming increasingly serious^56^, geological relics of exploitation and management should be based on the protected (Fig. 11). In addition to retaining the mining land and protected area of geological relics distributed on the construction land, respectively, the rest is classified into the corresponding land types. With the industrial development, the groundwater environment is suffered serious damage^57^, it directly relates the groundwater quality in protected area of groundwater resources and the local drinking water safety. The contamination of groundwater with hidden and difficult control properties, so the groundwater resources area should be protected in advance (Fig. 12). This study directly divides the type of groundwater source into an ecological land buffer.

## Results and Discussion

**Figure 13:**
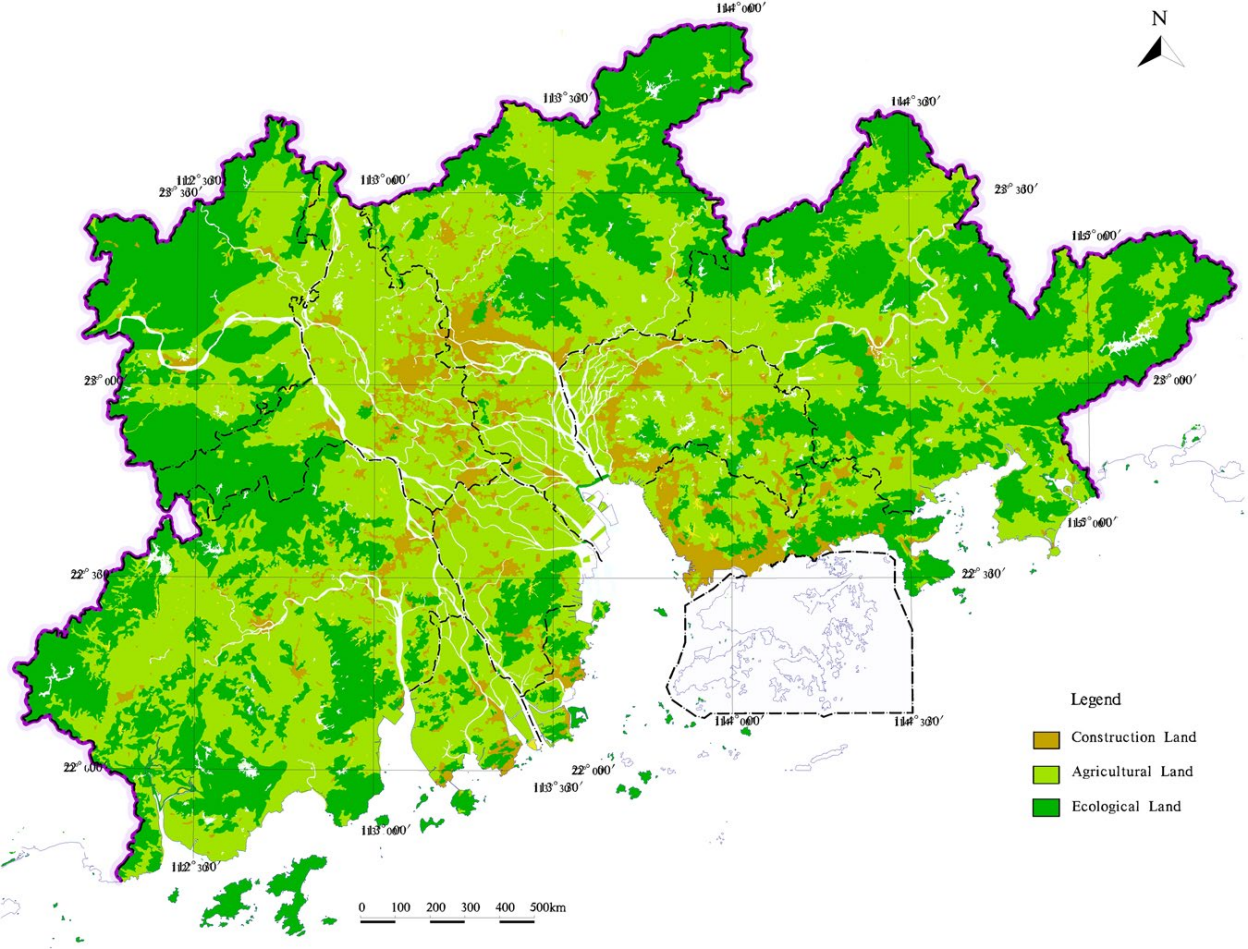
The zoning of land utilization in the study area.

According to the determination steps of land utilization and Fig. 3, the zoning results of different land utilization types are superimposed by method of MapGIS platform, and the result of land utilization zoning is got in the study area (Fig. 13).

**Figure 14:**
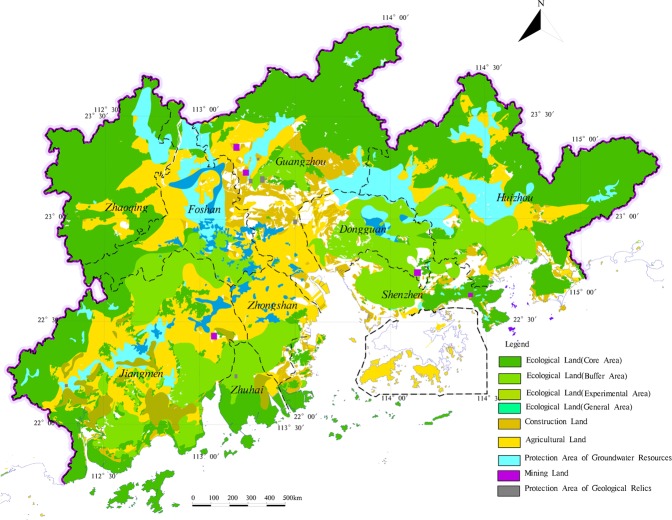
The comprehensive zoning of land utilization in the study area.

Based on the result of the land utilization zoning, the study analyses the distribution characteristics of the land utilization types, considered the suitability distribution of different land utilization types, the actual conditions, the regional function orientation and the requirement of ecological construction, the comprehensive zoning of land utilization in the study area is got (Fig. 14).

Agricultural land is widely distributed in areas with flat terrain within the study area. The low hills surrounding the study area, the terrain has a large slope, less agricultural land; The land with high agricultural suitability in the study area is mostly distributed in Jiangmen City and Huizhou City. The terrain has small slope, superior climatic conditions, good water quality conditions, superior location conditions, a certain agricultural foundation and suitable for large-scale mechanized operation of agriculture. For some moderate suitable agricultural land distribution in the northern part of the study area, due to scattered distribution and difficult development, on the premise that it cannot be converted into construction land, it should be classified as ecological land. In Guangzhou City, Foshan City and Dongguan City, where economic development is high, water and soil are highly polluted, and they are more easily absorbed by crops, thus affecting human health. At the same time, the population density in this area is highly concentrated, the per capita construction land area is low, part of the agricultural land in this region is divided into construction land to alleviate the tension between the supply and demand of the people in the study area. In addition, to maintain the stability of the ecosystem within the study area, some of the circular peripheral ecological core areas are occupied by agricultural land under realistic conditions, such areas should actively implement the policy of “returning farmland to forests” to guide the transformation of agricultural land to ecological land.

## Conclusions

Rapid urbanization has brought serious geological environmental problems, land resource utilization seriously restricts social development. Taking Pearl River Delta Economic Zone as an example, a variable weight-analytical hierarchy process-comprehensive index evaluation model was proposed to land utilization types zoning. In this process, based on 1:250000 Pearl River Delta Geological Survey Data, the model takes ecological protection zoning for the study area from assessing the importance of ecological service function in four aspects: biodiversity, water conservation, soil conservation and coastal zone protection. Based on this, the evaluation index system was established from the perspective of geological environment to evaluate the suitability of agricultural land and construction land. And re-zoning the type of land that has a special effect on the socio-economic and the ecology. According to the actual land utilization status and land suitability distribution, the results of land utilization zoning is got. The results showed that the area of ecological land, agricultural land and construction area in the study area accounted for 64.8%, 25.1%, and 10.1% of the total area of the study area, respectively.

Overall, the results show that the current land utilization in the Pearl River Delta is unreasonable because it does not consider geological environmental conditions, resulting in a decline in ecological service functions. This study provides an experience for regional zoning of multiple land utilization types. The method proposed in this paper, which applies future land utilization zoning in the study area and it can be recommended to conduct regional zoning for deterioration of ecological environment and the intensification of conflicts between people and land.

## Data Availability

The relevant datasets in this study are available from the corresponding author on reasonable request.
